# Items of General Interest

**Published:** 1915-07

**Authors:** 


### Items of General Interest.

---

Dr. J. G. Harrison will establish a sanitarium at McAllen for the lower Rio Grande valley.

---

Positive cure of tetanus by the simple injection of the juice of cactus leaf was a discovery related to the members of the American Medico-Pharmaceutical League at the Astor recently by Dr. D. B. DeWaltoff, of New York.

---

The China Medical Board recently established by the Rockefeller Foundation for the purpose of improving medical and hospital conditions in China gives promise of doing incalculable good in a country where, aside from the medical missionaries, there have been very few representatives of modern medicine.

---

An elixir to relieve fatigue has been discovered by Prof. Walter B. Cannon, of the Harvard Medical School. The use of adrenin, a substance found in the human body and also in sheep and calves, will accomplish in five minutes what in two hours of rest is ordinarily required to do.

---

The discovery of a new treatment for tetanus, which is believed will be of great importance in decreasing mortality on European battlefields, has been made public by the Rockefeller Institute. Dr. S. J. Meltzer discovered that a solution containing Epsom salts, injected into the membranes of the spinal cord, had the effect of producing complete relaxation of the muscles of the jaw for several hours.

---

Dr. W. C. Rucker, Assistant Surgeon General of the Public Health Service, after outlining the danger to the United States from plague germs carried half way across the world by rats in ships, urged co-operation of every medical man in affected territories to eliminate this menace to the United States. He vigorously condemns the "half-hearted" interest shown by the health officers of the United States, as well as foreign countries.

---

The University of Texas has kindly consented to establish a summer normal for the benefit of the county and city health officers of the State as well as all other physicians and teachers who will attend. The terms will be three weeks, beginning July 14, 1915. The program will consist of lectures on municipal health, organization and administration of public health, public health laws, vital statistics, anti-narcotic law, human carriers of disease, epidemiology, purification of water supplies, plumbing and sewers, septic tanks, vaccination, rabies and its treatment, infant feeding, baby welfare, sex hygiene, laboratory diagnosis, medical supervision in public schools, serums and bacterines, rural homes, school hygiene and sanitation, rural health problems and pure foods.

---

Feminine idealism must be enriched by racial idealism. Humanity is struggling upward.—*Medical Review of Reviews.*

